# Commentary: Reproducibility in Psychological Science: When Do Psychological Phenomena Exist?

**DOI:** 10.3389/fpsyg.2017.00918

**Published:** 2017-06-09

**Authors:** David Trafimow

**Affiliations:** Department of Psychology, New Mexico State UniversityLas Cruces, NM, United States

**Keywords:** non-existence, reproducibility, replication, auxiliary assumptions, verification, falsification

Iso-Ahola (2017) comments about the questionability of reproducibility and of attempting to demonstrate the non-existence of psychological entities. For full disclosure, I was a reviewer. I agree with much of what Iso-Ahola states but nevertheless have some reservations about this well-argued piece. My reservations do not concern ego-depletion, but are more general.

Iso-Ahola correctly indicates the importance of auxiliary assumptions in the disconfirmation of theories. Researchers can blame empirical defeats (the prediction derived from the theory does not work out empirically) on the theory or on auxiliary assumptions. Consequently, an empirical defeat does not necessarily provide a strong case against the theory or existence of a theoretical entity. But Iso-Ahola's strong emphasis on the limitations of negative evidence come at the cost of underestimating the limitations of positive evidence. It is important to bring out that auxiliary assumptions can be used to explain away empirical victories, just like empirical defeats (Trafimow, 2003, 2017). Therefore, empirical victories need not be more definitive than empirical defeats; such judgments should be made on a case-by-case basis.

But if empirical victories do not have a special status over empirical defeats, where does this leave the argument against the importance of empirical defeats? Given the possibility of attributing experimental findings, whether these are empirical victories or empirical defeats; to the conjunction of auxiliary assumptions and the proposed theory, or to the conjunction of auxiliary assumptions and a competing theory (whether it has or has not been stated yet); there is no a priori reason to take empirical victories more seriously than empirical defeats. If one refuses to take empirical defeats seriously, as in the Iso-Ahola scheme, then intellectual completeness and consistency require one not to take empirical victories seriously either. It is problematic to take empirical victories as favoring the existence of psychological constructs, while at the same time refusing to take empirical defeats as disfavoring their existence.

Another issue pertains to variability in human behavior and the alleged negative consequences for replicability that I believe Iso-Ahola overstates. Most experiments in social psychology are one-shot studies where there are different participants in the original experiment and in the replication experiment, and so intra-individual variability is less important than Iso-Ahola seems to think. As an analogy, consider 4,000 coins. The behavior of each coin is highly variable with respect to landing heads or tails, across trials. Nevertheless, we might divide the coins into two experiments, with 2,000 coins in each experiment. We would expect ~1,000 heads in Experiment 1, and also in Experiment 2. Although we might argue about what precisely counts as a replication (e.g., 975–1,025 heads in both experiments; 970–1,030 heads in both experiments, and so on), it would be a good bet that the two experiments would replicate at an approximate level. The fact that each coin is highly variable in its individual behavior does not prevent the conduction of a replicable experiment. Even if there were much inter-coin variability in probability of heads, reproducible findings nevertheless can be obtained provided random selection of coins from the population of coins. Moving to humans, the mere fact that human behavior is highly variable is not an insurmountable barrier to replication, though the sample sizes researchers typically use doubtless are insufficient.

I will end with my favorite experiment from the history of physics—the Michelson-Morley experiment (1887), which was a resounding empirical defeat (Michelson and Morley, 1887). In the 1880s, physicists believed that the universe was filled with the luminiferous ether that provided the medium for light waves to reach Earth from the stars. Michelson and Morley invented an interferometer to detect the luminiferous ether and failed to detect it. Physicists accepted that although one cannot prove or disprove, in absolute terms, the existence, or non-existence of theorized entities, such as the luminiferous ether, the empirical defeat provided an exceptionally strong case against the existence of the luminiferous ether. This empirical defeat was one of the greatest and most crucial experiments in the history of physics. Michelson received a Nobel Prize in 1907. (For an earlier and reproducible example of non-existence, Lavoisier disconfirmed the existence of phlogiston).

At the population level, the effect size is zero. But at the sample level, Carver's reanalysis of the Michelson-Morley data resulted in a miniscule sample effect size, but one nevertheless sufficiently large to obtain a statistically significant finding because of the many data points (Carver, 1993). There is insufficient space here to explain why the null hypothesis significance testing procedure is invalid, but one of its many disadvantages is that it causes researchers to think of replication in terms of obtaining statistical significance in two experiments. If we accept the usual physics interpretation that there is no luminiferous ether, and so the population effect size is zero, then obtaining effect sizes near zero in subsequent experiments renders them successful replications. Or at least this would be so if psychologists could get statistical significance out of their minds.

In the end, we must decide whether or not we wish psychology to be a science. If so, it follows that empirical victories and defeats must be reproducible, though much fun can be had arguing about precisely what we mean by “replicate.” In contrast, to the extent that we fail to insist on reproducible findings, it becomes increasingly difficult to distinguish psychology from religion, philosophy, and so on. Psychology is not physics, and there are problems that are unique to the social sciences, but that is no justification for abandoning the requirement that findings be replicable, nor failing to keep an open mind that some proposed entities might not exist even if published in top journals, any more than that the luminiferous ether or phlogiston exist (to the best of current knowledge, of course). As Trafimow and Rice (2009) demonstrated, there is much that is wrong with how psychologists conduct their research and even think about their research, that can be improved dramatically. Making the improvements is to be preferred over questioning whether we can do it.

## Author contributions

The author confirms being the sole contributor of this work and approved it for publication.

### Conflict of interest statement

The author declares that the research was conducted in the absence of any commercial or financial relationships that could be construed as a potential conflict of interest.
